# Oleanolic acid–chitosan compound inhibits mitochondrial autophagy and malignant transformation of lung cancer through the PTEN/AKT pathway

**DOI:** 10.55730/1300-0144.6163

**Published:** 2025-09-23

**Authors:** Abulimiti ABULAITI, XiaoHong SUN, Waresijiang YIBULAYIN, Dan HE, KeMing XU, Xiayimaierdan YIBULAYIN

**Affiliations:** Department of Thoracic Surgery Ward I, The Affiliated Cancer Hospital of Xinjiang Medical University, Urumqi City, China

**Keywords:** Oleanolic acid-chitosan compound, PTEN/AKT signaling pathway, mitochondrial autophagy, apoptosis

## Abstract

**Background/aim:**

Oleanolic acid–chitosan compound (OAC) shows potent antinonsmall cell lung cancer (NSCLC) activity, but its mechanisms remain unclear. This study elucidates how OAC modulates autophagy and apoptosis via the Phosphatase and Tensin Homolog/Protein Kinase B (PTEN/AKT) signaling pathway.

**Materials and methods:**

Oleanolic acid was coupled with chitosan to synthesize OAC. A549 and MRC-5 cells were cultured in Dulbecco’s Modified Eagle Medium and Minimum Essential Medium, respectively, supplemented with 10% fetal bovine serum and penicillin–streptomycin. Cells were pretreated with chloroquine before OAC treatment. Cell viability was assessed using the CCK-8 assay, while 5-bromo-2′-deoxyuridine (BrdU) and colony formation tests were employed to evaluate cell proliferation. Apoptosis was measured by flow cytometry using Annexin V-FITC/PI (fluorescein isothiocyanate/propidium iodide) staining. Autophagy was monitored through western blot analysis of LC3, SQSTM1, Atg5, Beclin1, and LAMP1, and confirmed by immunofluorescence and transmission electron microscopy (TEM). Mitochondrial membrane potential (MMP) was determined by JC-1 staining, and acridine orange staining was used to detect acidic vesicular organelles (AVOs). Additionally, PTEN overexpression was induced by transfecting cells with the hemagglutinin tag (HA)-PTEN plasmid.

**Results:**

OAC treatment significantly inhibited cell viability and proliferation in A549 cells, as evidenced by decreased CCK-8 absorbance, reduced BrdU incorporation, and fewer colony formations. Flow cytometry revealed a marked increase in apoptotic cells following OAC treatment. Western blot analysis demonstrated altered expression levels of apoptosis-related proteins (Bcl-2, Bax, Cytochrome c, caspase-9, caspase-3, PARP) and autophagy markers (LC3, SQSTM1, Atg5, Beclin1, LAMP1). Immunofluorescence and TEM further confirmed the induction of autophagy. AO staining showed increased AVOs, while JC-1 staining indicated a reduction in MMP. PTEN overexpression enhanced OAC-induced apoptosis and autophagy.

**Conclusion:**

OAC effectively inhibits cell proliferation and induces apoptosis and autophagy in A549 cells, with PTEN playing a regulatory role in these processes. These findings suggest that OAC may serve as a potential therapeutic agent for cancer treatment.

## 1. Introduction

Lung cancer is estimated to have caused 2.2 million deaths worldwide in 2020, with a mortality rate of 60%–80% [1]. Lung cancer can be divided into different types based on histological heterogeneity, with nonsmall cell lung cancer (NSCLC) being the most common, comprising 84% of all lung cancer cases [2–4]. Patients with advanced NSCLC usually receive surgery, radiotherapy, and chemotherapy as part of their treatment plan. For patients with advanced NSCLC, dual platinum therapy is currently the standard first-line treatment. Due to poor selectivity, serious side effects, and drug resistance, several patients are unable to receive satisfactory treatments [5–7]. Therefore, alternative treatments for NSCLC are urgently needed.

Oleanolic acid, a pentacyclic triterpenoid, is present in oleaceous plants. It is primarily located in the epidermis, where it serves as a defense against microorganisms. Oleanolic acid has antiinflammatory and antioxidant properties and has been isolated from about 1620 plants [8]. It is believed to have antiinflammatory, antioxidant, hepatoprotective, antitumor, antiviral, and antidiabetic activities [9–13]. Oleanolic acid has been shown to induce arrest of the S and G2/M phases of pancreatic and lung cancer cells [14,15]. Chitosan nanoparticles are synthesized and coupled with oleanolic acid to obtain oleanolic acid–chitosan compound (OAC). Despite this, the mechanisms underlying OAC’s ability to combat NSCLC remain poorly understood.

Autophagy is a self-degradation process that breaks down proteins and damaged or excessively degraded organelles by creating double-membrane autophagosomes [16]. Drugs, nutrient deficiency, hypoxia, oxidative stress, and hypoxia are some of the stimuli that cause autophagy [17,18]. The “double-edged sword” of drug-induced autophagy in cancer treatment lies in its ability to favor cellular survival [19] or cause autophagic death [20]. The Phosphoinositide 3-Kinase/Protein Kinase B/Mechanistic (or Mammalian) Target of Rapamycin (PI3K/AKT/mTOR) and the Phosphatase and Tensin Homolog/Protein Kinase B (PTEN/AKT) signaling pathways are classical pathways that regulate autophagy [21,22].

The study was designed to examine the possible mechanism of OAC in NSCLC and proposed a potential way OAC could fight NSCLC, demonstrating its worth as a therapeutic agent.

## 2. Materials and methods

### 2.1. Compounds

Oleanolic acid and chitosan were coupled [26]. Oleanolic acid (20 mL) was dissolved in anhydrous dimethyl sulfoxide by stirring. This mixture was then mixed with an acetic acid solution of 0.5% (w/v) chitosan (0.1 M, pH 4.7) at room temperature in the dark for 12 h. A pH of 9.0 was achieved by adding 1 M NaOH, and the solution was centrifuged at 2500 rpm. The sediment was then dialyzed in phosphate-buffered saline (PBS) (pH 7.4) for three days and in water for four days. Finally, the product was isolated and lyophilized [6].

### 2.2. Cell culture

A549 cells were placed in Dulbecco’s Modified Eagle Medium (DMEM), while MRC-5 cells were cultured in Minimum Essential Medium (MEM) under 5% CO_2_ at 37 °C. Both media were supplemented with 10% fetal bovine serum (Gibco, Waltham, MA, USA) and 10U/mL penicillin–streptomycin. Cells were treated with the autophagy inhibitor chloroquine (CQ; Sigma-Aldrich) at 10 mM for 24 h before treatment.

### 2.3. CCK-8

After overnight incubation in 96-well plates, cells were treated with drugs for 24 h, and 10 mL/well CCK-8 (Dojindo, Japan) was added for two to four h after treatment. The absorbance at 450 nm was measured on a microplate reader (Bio-Rad, Hercules, CA, USA).

### 2.4. BrdU test

The BrdU test was done according to the instructions of the BrdU Cell Proliferation ELISA kit (Abcam, UK). The duration of treatment with the drug and BrdU was 24 h and 12 h, respectively. Optical density was measured using a microplate reader.

### 2.5. Colony formation test

The cells were treated with OAC for 14 days after being inoculated into the six-well plate. Clones exceeding 50 cells were counted under a microscope following fixation in four percent paraformaldehyde and staining with crystal violet.

### 2.6. Flow cytometry

Apoptosis was evaluated using the FITC Annexin V Apoptosis Detection Kit (BD Biosciences, USA). The cells were reconstituted in 500 μL of one × binding buffer and stained with five μL of the Annexin V-FITC solution and five μL of the PI solution, respectively. Following a 15-min interval, the percentage of apoptotic cells was quantified using FACScan flow cytometry (BD Biosciences).

### 2.7. Western blot analysis

The cells were washed with precooled PBS, then lysed with lysis buffer (Beyotime, Shanghai, China) on ice for 20 min. The Bradford assay (Bio-Rad, USA) was employed to measure protein levels. Subsequently, the proteins were separated by 15% sodium dodecyl sulfate–polyacrylamide gel electrophoresis (SDS–PAGE) and transferred to polyvinylidene fluoride membranes. Proteins were blocked with five percent skim milk powder for one h at ambient temperature, followed by three washes with Tris-Buffered Saline with Tween 20 (TBST). The primary antibodies LC3 (ab192890), SQSTM1 (ab109012), Atg5 (ab108327), Beclin1 (ab302669), LAMP1 (ab278043), Bcl-2 (ab182858), Bax (ab192890), Cytochrome c (Cyt c, ab133504), caspase-9 (ab32539), caspase-3 (ab32351), PARP (ab32064), PTEN (ab267787), AKT (ab38449), p-AKT (ab18785), and GAPDH (ab8245) were incubated overnight at four °C. Abcam provided all the above antibodies. After TBST washing, the secondary antibody (CST, USA) bound to the corresponding horseradish peroxidase was incubated at 37 °C for one h. Proteins were then developed with an enhanced chemiluminescence kit (UltraSignal, China).

### 2.8. Immunofluorescence

Cells were fixed, permeated, and blocked. After overnight incubation with LC3 or LAMP1, goat antirabbit IgG coupled with horseradish peroxidase was incubated for one h, and 4′,6-diamidino-2-phenylindole was stained for 20 min. Images were captured using a laser-scanning confocal microscope (FV1000, Olympus, Tokyo, Japan).

### 2.9. Transmission electron microscopy (TEM)

After trypsinization, A549 cells were fixed in a mixture containing two percent glutaraldehyde and one percent tannic acid, incubated overnight at four °C in 0.1 M sodium carboxylate (pH 7.3), then rinsed three times with sodium acetate buffer, and incubated for two h in two percent osmium tetroxide buffer. Subsequently, the cells were submerged in one percent uranyl acetate for 15 min, centrifuged in three percent agarose at 45 °C, and chilled. The agarose blocks were dehydrated using a gradient of acetone, then embedded in Spurr resin and polymerized overnight at 65 °C. Subsequently, Slices measuring 80 nm were cut with the Reichert-Jung Ultracut E (20132; Leica Microsystems) ultramicrotome, stained with uranyl acetate and bismuth subnitrate, viewed under a Philips CM-10 transmission electron microscope (20122; FEI Italia), and captured on Kodak 4489 film.

### 2.10. Acridine orange (AO) staining

Cells were implanted in the six-well plate overnight, treated with OAC for 24 h, and stained with one μg/mL AO for 15 min. After PBS washing, cell analysis was performed using flow cytometry.

### 2.11. Mitochondrial membrane potential (MMP) assay

JC-1 staining was performed using the JC-1 MMP kit (Beyotime, Shanghai, China). Cells were first stained with the JC-1 solution, then washed with the JC-1 staining buffer. Carbonyl cyanide m-chlorophenyl hydrazone was taken as the positive control.

### 2.12. PTEN overexpression transfection

HA-PTEN overexpression plasmid (GeneCopoeia, USA) was transfected into cells using Lipofectamine 2000 and analyzed by western blot.

### 2.13. Statistical analysis

Data were expressed as mean ± standard error of the mean. SPSS Statistics 20 was applied for statistical analysis. Statistical differences were determined using an independent-samples t-test or one-way analysis of variance, with * p < 0.05, ** p < 0.01, and *** p < 0.001 indicating significance. At least three repetitions of each experiment were conducted.

## 3. Results

### 3.1. OAC inhibits NSCLC cell proliferation

**Figure 1:**
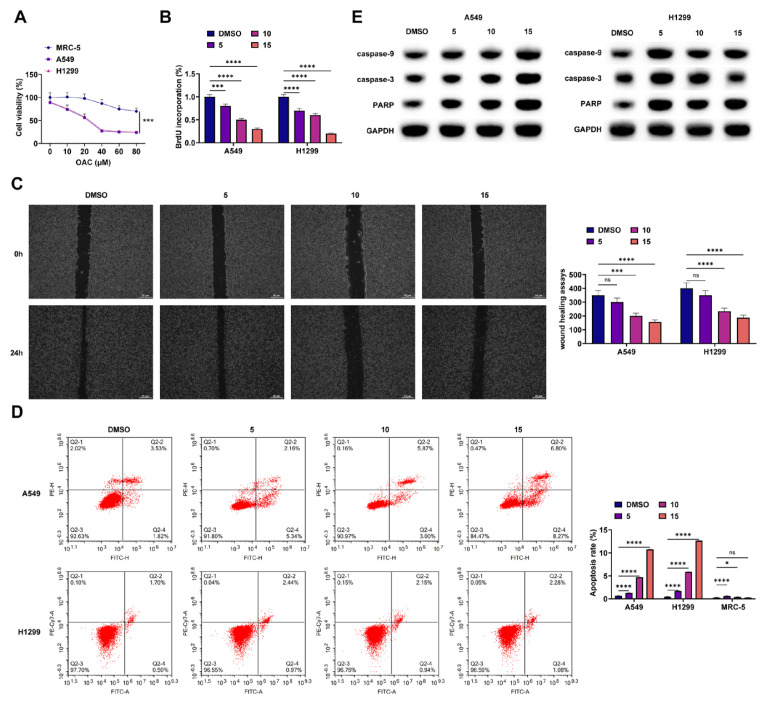
OAC inhibits NSCLC cell viability and growth. A. CCK-8 assay detected cell viability; B–C. BrdU and wound healing assays analyzed cell proliferation; D. Flow cytometry detected apoptosis; E. Western blot detected caspase-9, caspase-3, and PARP proteins.

The study focused on examining OAC’s cancer-fighting properties in NSCLC cells and its safety against normal lung cells, examining its impact on the survival of A549 and the normal lung cell line, MRC-5. The CCK-8 test revealed that OAC diminished the viability of NSCLC cells in a dose-responsive manner, yet minimally impacted the viability of MRC5 cells (Figure 1A). OAC inhibited NSCLC cell proliferation as confirmed by BrdU and colony formation assays (Figure 1B–C). According to flow cytometry results, OAC triggered apoptosis in NSCLC cells in a dose-dependent manner but had little effect on MRC-5 cells (Figure 1D). Western blot results demonstrated that OAC enhanced caspase-9, caspase-3, and PARP protein levels (Figure 1E). These data suggest that OAC is cytotoxic and induces apoptosis in NSCLC cells in a caspase-dependent manner.

### 3.2. OAC induces autophagy in NSCLC cells

**Figure 2:**
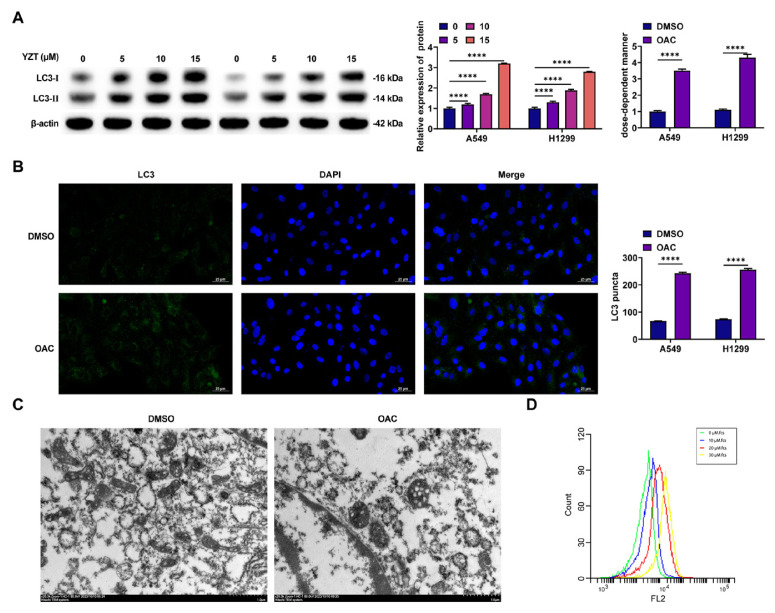
OAC induces autophagy in NSCLC cells. A. Western blot detected LC3; B. Immunofluorescence observed endogenous LC3 spots; C. TEM observed the ultrastructure of autophagosomes/autolysosomes; D. AO staining detected the formation of AVO.

Autophagy is a key mechanism by which cancer-inhibiting drugs act [23]. LC3-II levels were increased dose-dependently by OAC in NSCLC cells by western blotting, indicating that OAC induced autophagy in these cells (Figure 2A). Additionally, immunofluorescence showed that OAC significantly increased LC3 levels (Figure 2B). Moreover, the morphology of autophagosomes was directly examined using TEM, which showed that cells post-OAC treatment exhibited the classical ultrastructural features of autophagosomal bilayer vacuoles (Figure 2C). AO staining demonstrated that OAC promoted the formation of acidic vesicular organelles (AVOs) in a dose-dependent manner, another characteristic of autophagy (Figure 2D). These results suggest that OAC induces autophagy in NSCLC cells.

### 3.3. OAC induces complete autophagy flux

**Figure 3:**
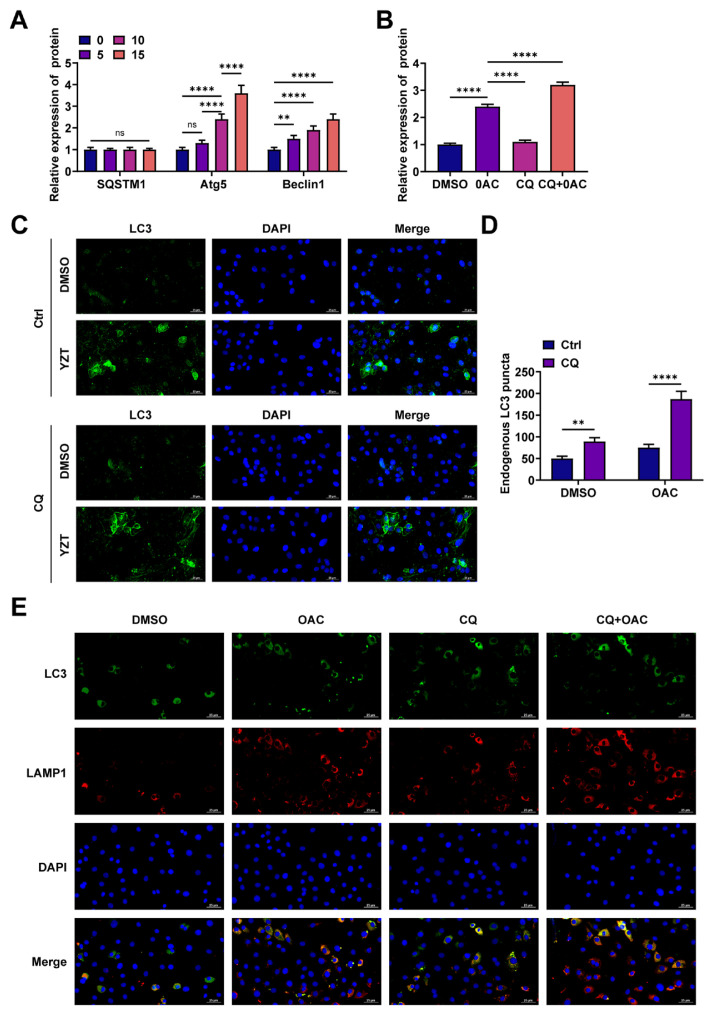
OAC induces complete autophagy flux. A. Western blot analysis of SQSTM1, Atg5, and Beclin1 levels; B. Western blot detected LC3-II levels; C–D. Monitoring endogenous LC3 spots by immunofluorescence (scale: five μm); E. Confocal microscopy localized LC3 and LAMP1 (Scale: 10 μm).

SQSTM1, Atg5, and Beclin1, which are involved in autophagy, were determined using western blots. Atg5 and Beclin1 were upregulated dose-dependently, but SQSTM1 did not show a significant difference with OAC treatment (Figure 3A). Moreover, the effect of the autophagy inhibitor CQ on LC3 levels was tested using western blotting. The findings indicated that LC3 levels were higher with OAC plus CQ than with OAC alone (Figure 3B). Observation of endogenous LC3 dots by immunofluorescence yielded similar results (Figure 3C–D). The process is tracked by immunofluorescence, with LC3 and LAMP1 colocalizing, as autophagosomes fuse with lysosomes during complete autophagy flux. Pearson analysis showed that CQ could promote the separation of LC3 and LAMP1; however, OAC promoted their overlap (Figure 3E).

### 3.4. Autophagy inhibition exacerbates OAC-mediated mitochondrial apoptosis

**Figure 4:**
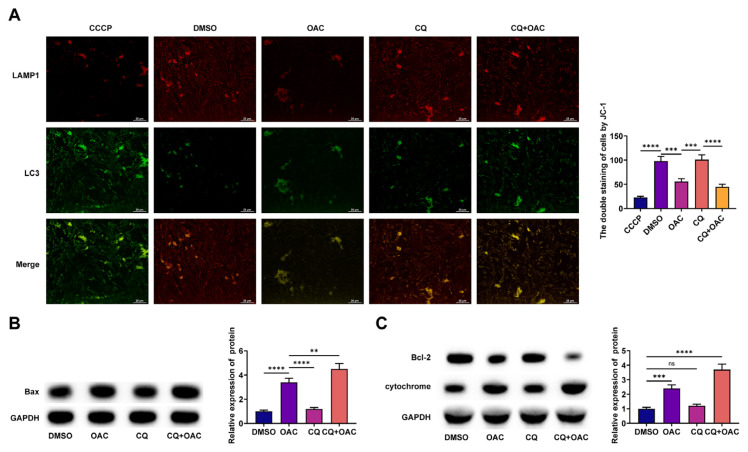
Autophagy inhibition exacerbates OAC-mediated mitochondrial apoptosis. A. JC-1 staining analyzed MMP; B–C. Western blot detected Bax/Bcl-2 ratio and Cyt c levels.

In mitochondrial apoptosis, MMP is reduced, and Cyt C is released into the cytoplasm. Mitochondrial depolarization was assessed using JC-1 staining, where the red/green fluorescence intensity ratio indicated MMP. CQ enhanced OAC-mediated downregulation of MMP (Figure 4A). Western blot confirmed that CQ accentuated OAC-mediated increase in Bax/Bcl-2 ratio and Cyt c levels (Figure 4B–C). Autophagy provides cytoprotection by counteracting OAC-mediated mitochondrial apoptosis.

### 3.5. OAC initiates autophagy via the PTEN/AKT axis

**Figure 5:**
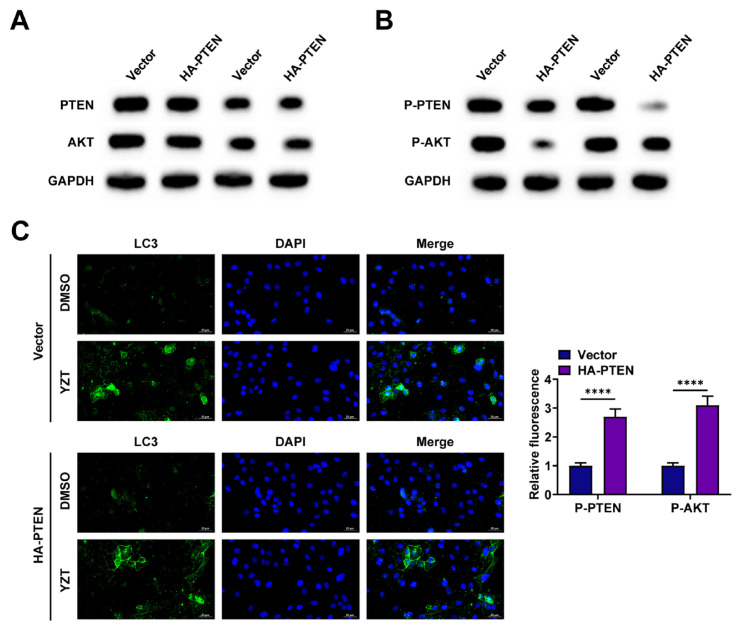
OAC initiates autophagy via the PTEN/AKT axis. Western blot-detected PTEN/AKT; B. Western blot analysis measured p-PTEN, p-Akt, and LC3-II; C. Detection of endogenous LC3 points by immunofluorescence (scale: five μm).

There is a classical negative regulation of autophagy through the PTEN/AKT axis [24,25]. Western blot showed that OAC reduced PTEN and AKT levels dose-dependently (Figure 5A). In PTEN-overexpressing cells, p-Akt and LC3 were measured by western blot to determine whether OAC induces autophagy through PTEN. Overexpression of PTEN partially reversed the OAC-induced downregulation of p-Akt and counteracted the OAC-mediated increase in LC3-II (Figure 5B). When PTEN was overexpressed, immunofluorescence revealed reduced LC3 spots, indicating that PTEN overexpression attenuated the OAC-induced increase in LC3 spots (Figure 5C).

## 4. Discussion

Inducing cell death as the sole strategy for cancer treatment is no longer effective. New treatment methods focus on the tumor microenvironment, managing immune and inflammatory responses, preventing angiogenesis, and addressing cancer cell death with selective compounds that minimize systemic toxicity. As a result, phytochemicals have been shown to benefit cancer in preclinical and epidemiological studies [26,27]. Due to their selectivity and high biological activity against cancer cells, triterpenoids are a promising multifaceted option for preventing and treating cancer [28]. Oleanolic acid exhibits an antiproliferation effect against cancers, including breast, gastric, and liver cancers [29–31]. Therefore, molecule-targeted therapy has gradually replaced traditional cytotoxic drugs in anticancer treatment. PI3K/3-Phosphoinositide-Dependent Protein Kinase-1 (PDK1)/AKT and PTEN/AKT signaling axes have emerged as key targets for cancer therapy. According to this study, OAC induced autophagy by negatively regulating the PTEN/AKT axis. Specifically, our results showed that OAC dose-dependently reduced PTEN and AKT levels, and overexpression of PTEN partially reversed the downregulation of p-Akt and the increase in LC3-II induced by OAC, directly confirming that the PTEN/AKT axis is the key signaling pathway by which OAC initiates autophagy in NSCLC cells. This finding enriches the understanding of the molecular mechanisms by which triterpenoids regulate autophagy and provides a new target for the development of NSCLC-targeted drugs.

Autophagy serves four roles: protective, nonprotective, cytotoxic, and cytoinhibition. Increased sensitivity to treatment and increased apoptosis are characteristics of cell-protective autophagy [32,33]. Current research indicates that autophagy is involved in a broader range of disease processes, including cancer, and is considered a mechanism of cell death [34,35]. In this study, CQ-blocking autophagy significantly enhanced OAC-mediated cytotoxicity and apoptosis, demonstrating that OAC-induced autophagy has a cytoprotective effect. This finding is consistent with previous studies in other cancer types. For instance, in a study on pancreatic cancer, inhibiting autophagy with CQ sensitizes cancer cells to chemotherapy, indicating that autophagy acts as a protective mechanism against the cytotoxic effects of the treatment [36]. Similarly, in breast cancer research, blocking autophagy enhances the efficacy of targeted therapies, further supporting the notion that autophagy can serve as a shield for cancer cells [37]. By inhibiting this protective autophagy, we can potentially achieve a more sensitive response to cancer treatment, making OAC a promising new drug candidate against NSCLC, with its anticancer effects being highly dependent on autophagy [38]. While CQ, Wortmannin, and Bafilomycin A1 are used to pharmacologically inhibit autophagy, genetic manipulation offers a different approach [39,40]. In this work, CQ was utilized because it is convenient and feasible in clinical practice.

Apoptosis usually involves both exogenous and endogenous apoptosis. The latter is characterized by the regulation of mitochondrial apoptosis by proapoptotic and antiapoptotic Bcl-2 family members. Activated Bax and BAK induce mitochondrial outer membrane permeability under proapoptotic stress [41]. Oleanolic acid can inhibit hepatocellular carcinoma progression by inducing mitochondria-mediated apoptosis and autophagy [42]. It has been proven that apoptosis induced by oleanolic acid derivatives is related to mitochondrial dysfunction [43]. In this study, compared with OAC alone, the combined use with CQ further reduced MMP, elevated the Bax/Bcl-2 ratio, and increased Cyt c expression, suggesting that autophagy induced by OAC plays a cellular protective role by antagonizing mitochondrial apoptosis induced by OAC. This aligns with the idea of the “autophagy-apoptosis interaction” in cancer cells, where autophagy can serve as a double-edged sword. On one hand, it supports cell survival during stress, such as nutrient deprivation or chemotherapy-induced damage, by recycling cellular components and generating energy. On the other hand, excessive autophagy can cause cell death, known as autophagic cell death. In our investigation, OAC-induced protective autophagy appears to serve as a survival mechanism for NSCLC cells, mitigating OAC-induced apoptosis. By inhibiting autophagy with CQ, this protective mechanism was disrupted, allowing the apoptotic pathway to proceed more effectively [44,45]. The outcomes of the study are consistent with the findings that autophagy regulates mitochondrial apoptosis and aids cell recovery from apoptosis [46,47]. In addition, our results from CCK-8, BrdU, colony formation assays, and flow cytometry showed that OAC can inhibit the proliferation and induce apoptosis of NSCLC cells in a dose-dependent manner, while having little effect on normal lung cells (MRC-5). This indicates that OAC shows a degree of selectivity in anti-NSCLC, which is an important advantage for its potential clinical application, as it can reduce toxicity and side effects in normal tissues.

In summary, OAC can act as a protective agent for NSCLC. This study is the first to demonstrate that OAC induces protective autophagy in NSCLC cells through the PTEN/AKT pathway. Our data provide new insights into the targeted pathways involved in NSCLC and highlight novel therapies for complexes that have not yet been adequately studied. Future studies should aim to clarify the specific molecular mechanisms underlying the interactions among OAC, autophagy, and apoptosis. Clinical trials are also needed to evaluate the safety and effectiveness of OAC in NSCLC patients, either alone or in combination with other therapeutic agents. Optimizing OAC utilization and understanding its mechanisms could improve NSCLC patient treatment outcomes, offering renewed hope against this disease. Therefore, it is hoped that OAC can be applied in clinical research to generate new ideas for its use in the clinical therapeutic targets of NSCLC.

## Data Availability

The datasets used and/or analyzed during the present study are available from the corresponding author upon reasonable request.
